# Exploring Barriers to Inclusivity: Systematic Analysis of Exclusion Criteria and Potential Bias in Clinical Cancer Trials for Psychiatric and Neurological Conditions in European Protocols

**DOI:** 10.1002/pon.70182

**Published:** 2025-05-12

**Authors:** Margherita Dahò, Veronica Coppini, Maria Vittoria Ferrari, Giulia Ferraris, Virginia Sanchini, Dario Monzani, Roberto Grasso, Chiara Agnello, Giuseppe Badalamenti, Laura Algeri, Gabriella Pravettoni

**Affiliations:** ^1^ Department of Psychology Educational Science and Human Movement University of Palermo Palermo Italy; ^2^ WeSearch Lab ‐ Laboratory of Behavioral Observation and Research on Human Development University of Palermo Palermo Italy; ^3^ Applied Research Division for Cognitive and Psychological Science IEO European Institute of Oncology IRCCS Milan Italy; ^4^ Department of Oncology and Hemato‐Oncology University of Milan Milan Italy; ^5^ Centre for Biomedical Ethics and Law Department of Public Health and Primary Care KU Leuven Leuven Belgium; ^6^ Department of Precision Medicine in Medical Surgical and Critical Care (Me.Pre.C.C.) Section of Medical Oncology University of Palermo Palermo Italy

**Keywords:** bias, cancer, clinical trials, disparities, healthcare, inclusivity, mental health, Oncology, Psycho‐oncology, vulnerable population

## Abstract

**Background:**

Cancer clinical trials often employ exclusion criteria that can impact vulnerable populations, particularly individuals with psychological, psychiatric, or neurological conditions.

**Aims:**

This study aimed to analyze the prevalence and nature of exclusion criteria in clinical trials for prostate, breast, and lung cancers.

**Methods:**

The EU Clinical Trials Register identified 51 protocols uploaded between 2022 and 2024. Thematic content analysis categorized exclusion criteria, and the justifications provided, while frequency analysis quantified their prevalence.

**Results:**

After excluding five protocols (two non‐English and three inaccessible), the final dataset comprised 46 protocols: 13 for prostate cancer (22.8%), 24 for breast cancer (42.1%), and 9 for lung cancer (15.8%). Exclusion criteria targeting vulnerable populations were present in 78.3% of protocols, categorized into five themes: *psychiatric conditions* (24.6%), *neurological conditions* (22.8%), *other psychological conditions* (22.8%), *legal/guardianship status* (5.3%), and *unspecified conditions* (24.6%). *Compliance concerns* (39.1%) were the most common justification, followed by *informed consent challenges* (32.6%), *safety risks* (13%), *drug interference* (10.9%), and *not in the best interest* (4.3%). Notably, 29.1% of protocols lacked justification for exclusions, raising ethical and transparency concerns.

**Conclusions:**

The exclusion of vulnerable populations may limit the inclusivity and generalizability of cancer research. Heuristic biases and systemic practices can potentially influence this. Exploring the role of these factors and considering adaptive trial designs, along with providing detailed justifications for exclusion criteria, could support more equitable and representative clinical research.

## 1 Introduction

Clinical cancer trials are essential for improving therapeutic strategies and patient outcomes, yet disparities in cancer care are evident in both routine healthcare and clinical research. Patients encounter systemic barriers, cultural stigma, and misinformation, leading to unequal access to treatment and varying outcomes [1, 2, 3]. These inequities are reflected in clinical trials, where vulnerable populations are often underrepresented. The International Council for Harmonization (ICH), which issues guidelines to standardize the interpretation and application of pharmaceutical approval standards, defines 'vulnerable participants' as individuals whose decision to enroll in a clinical trial may be unduly influenced by expected benefits or fear of repercussions from authority figures [4]. These include individuals in hierarchical systems (e.g., medical students, military personnel, detainees) and others facing barriers to informed consent (e.g., minors, minorities, refugees, nursing home residents, etc.). Therefore, at least in this Document, as well as in other policy guidelines, the concept of vulnerability is closely tied to the ability to give free and informed consent [5]. Notably, some key ethical frameworks do not explicitly address vulnerability, given that they focus solely on the lack of capacity to provide informed consent as the basis for special protective measures. For instance, the Council of Europe's Convention on Human Rights and Biomedicine, known as the Oviedo Convention, limits its protective provisions to ‘*persons not able to consent to research’* ([6], art. 17).

The underrepresentation of vulnerable populations is thus mainly due to exclusion criteria that, while intended to ensure safety and protocol adherence, ultimately perpetuate significant disparities in both research participation and evidence base for vulnerable groups [7, 8, 9]. Specifically, Shepherd et al. [10], in a systematic review of clinical trials, highlighted the broader issue of excluding individuals who cannot provide informed consent, demonstrating that adults lacking the capacity to consent are routinely excluded from clinical trials in the UK. Their findings raise concerns that ethical safeguards may, in practice, function as barriers to inclusion rather than as mechanisms of protection [10]. Building on this, recent scholarship has further illuminated how governance measures, such as ethics codes and research approval processes, can inadvertently lead to the unfair exclusion of vulnerable populations. For example, Schroeder, Chatfield, Chennells, Partington, Kimani, Thomson, and Louw [11] analyzed 57 research ethics codes and guidelines and found that protective measures, though well‐intentioned, are often applied in a blanket fashion that fails to account for the diversity and context‐specific nature of vulnerability. This scenario is prevalent in psychiatric and neurological conditions, where such impairments may influence the ability to provide informed consent or to comply with the study. As a result, excluding these individuals from clinical trials restricts access to potentially beneficial treatments and creates a significant knowledge gap concerning the efficacy and safety of new therapies [12, 13]. However, despite ethical and practical challenges, there is a growing recognition of the need to include underrepresented groups in clinical research [8, 9, 11, 14, 15]. Indeed, excluding individuals with mental conditions may result in less effective or accessible treatments, further exacerbating healthcare disparities [11, 13].

### 1.1 The Current Study

Addressing the concerns surrounding the inclusion of individuals with psychiatric or neurological conditions in cancer trials necessitates a comprehensive understanding of the specific barriers they face. A critical evaluation of the reasons researchers provide for excluding participants is essential to determine whether these justifications are well‐substantiated and to identify any potential unfairness in the exclusion process. It is also important to assess any unjustified exclusions, as they may lead to inequitable treatment. This study thus focuses on protocol‐level decisions and aims to investigate the extent and implications of excluding individuals with psychiatric or neurological disorders from cancer clinical trials, analyzing the reasons provided for such exclusions and their alignment with ethical and scientific justifications. We seek to emphasize the importance of promoting inclusive research practices and propose exploring modifications in trial design and regulatory frameworks to enhance equitable participation and strengthen the evidence base for treating cancer among diverse patient populations.

## 2 Methods

### 2.1 Protocols Search

We searched for clinical trials on the EU%20Clinical%20Trials%20Register, a comprehensive database covering interventional trials in EU member states and the European Economic Area. Since the implementation of the EU Clinical Trials Regulation (Regulation EU No 536/2014), recording clinical trials in this database has been mandatory, enhancing data completeness and reducing publication bias. This ensures a broad representation of studies, including those with less favorable or inconclusive results, making the Register a vital resource for researchers, policymakers, and healthcare professionals. The mandatory registration strengthens the reliability and relevance of our analysis, providing a transparent and comprehensive view of the European clinical research landscape.

Between May 20‐27, 2024, we selected clinical trials on lung, prostate, and breast cancers from the EU Clinical Trials Register. Lung cancer is the top cause of cancer‐related deaths globally, while prostate and breast cancers are the most diagnosed cancers in men and women, highlighting the significant impact these illnesses have globally [16, 17, 18]. Therefore, advancements in their treatment could have a broad public health impact. Additionally, these cancers are the focus of extensive research, providing a rich dataset that strengthens the robustness of our analysis.

Using the advanced search criteria within the Register, M.D., V.C., M.V.F., and G.F. specified the pathology to filter the trials accordingly and downloaded the protocols. No specific timeframe for exclusion was established, ensuring that the analysis captured a comprehensive and up‐to‐date representation of protocols. All interventional trials with available protocols for the cancer types selected were included in the analysis. Given the focus on eligibility criteria—particularly exclusions related to psychiatric and neurological conditions—trials were not classified based on clinical phase, type of intervention, or therapeutic aim. M.D., V.C., and M.V.F. then performed a keyword search within the protocols to identify exclusion criteria. Keywords included “excl‐” (to capture terms like exclusion criteria, exclusive, excluding, etc.), “mental,” “psychiatric,” and “psych‐” (to highlight terms related to all psychological aspects), “neuro‐” or “cognit‐” (to identify terms related to neurological or cognitive conditions), and “vuln‐” (to find terms related to vulnerability). Subsequently, the inclusion and exclusion criteria, including the sub‐paragraph sections, were carefully reviewed. This approach allowed us to determine whether these population groups were excluded from the clinical trials. In our analysis, we included all psychiatric conditions, which refer to mental health disorders affecting mood, thinking, and behavior. We also considered neurological conditions, encompassing disorders of the central nervous system. Furthermore, we analyzed exclusions based on patients' medications. Notably, several protocols excluded specific medications—such as antipsychotics, antidepressants, and anticonvulsants—without explicitly mentioning mental or neurological conditions. In these cases, the protocols were categorized based on the excluded medications, such as placing exclusions of antipsychotic users within the psychiatric disorder category.

### 2.2 Data Analysis

The search for clinical trials, exclusion criteria, and their justifications was directly transcribed from the protocol into Excel. Then, M.D. and D.M. analyzed the data by employing a mixed‐method approach, which involved assessing both the frequency of word occurrences and the specific terminology used to build coherent clusters. For the thematic analysis, we adhered to Clarke and Braun's recommendations [19], conducting the analysis manually without using specialized software. First, the entire dataset was reviewed several times to gain a comprehensive understanding, and notes were made to highlight significant ideas. A systematic coding process was applied, assigning labels to data segments to capture their core meaning. Major themes were grouped into clusters based on similarity or category and further refined by curacy. Each cluster was finally labeled according to the dominant themes identified during the analysis and discussed with all authors.

Finally, a content analysis using SPSS identified the most frequently occurring themes. This process allowed us to examine the prevalence and distribution of the exclusion criteria and their justification across the different types of cancer clinical trials, providing a comprehensive understanding of the patterns observed. The final stage involved discussing the clusters with all authors to construct a cohesive narrative that effectively addressed the research questions.

## 3 Results

### 3.1 Protocol Search

The protocol search identified 51 cancer clinical trials across three specific cancer types—prostate, breast, and lung cancer—using the EU Clinical Trials Register. These trials were selected based on their relevance to the study's focus and were all uploaded between 2022 and 2024, ensuring the dataset reflected the most recent advancements in clinical research. The initial pool comprised 14 prostate cancer trials, 26 breast cancer trials, and 10 lung cancer trials. Trials unavailable in English or inaccessible for download were excluded. The final dataset comprised 46 clinical trials: 13 for prostate (22.8%), 24 for breast (42.1%), and 9 for lung cancer (15.8%). All data can be found in the Supporting Information S1: (Table A).

### 3.2 Exclusion Criteria

Findings reveal that exclusion criteria related to vulnerable populations, particularly those with psychological, psychiatric, or neurological conditions, are commonly found in clinical trials. Among the analyzed protocols, 36 (78.3%) reported such exclusion criteria. In contrast, 10 protocols (21.7%) did not specify any exclusion criteria, suggesting that these populations were not excluded. Thematic analysis identified 5 principal categories of exclusion criteria based on recurring themes. Below are the identified clusters.

1. *Psychiatric Conditions* (24,6%): This category includes individuals with psychotic symptoms, such as those diagnosed with schizophrenia or bipolar disorder. Examples include statements like: “*History of significant neurological or psychiatric disorders including psychotic disorders, dementia or seizures”* (protocol 26).
2. *Neurological Conditions* (22,8%): This group covers a wide range of cognitive disorders, including seizures, Parkinson's, Alzheimer's, dementia, and other cognitive disabilities related to central nervous system pathologies. Although often grouped with psychiatric disorders, they were categorized separately due to distinct concerns. Examples include: “*Patients with a history of relevant CNS pathology or current relevant CNS pathology* (e.g.*, seizure, paresis, aphasia, cerebrovascular ischemia/hemorrhage, severe brain injuries, dementia, Parkinson's disease, cerebellar disease, organic brain syndrome, psychosis, coordination or movement disorder*)” (protocol 4).
3. *Other Psychological Conditions* (22,8%): This category encompasses mental health conditions that are not strictly categorized as psychiatric disorders, yet are still significant enough to justify exclusion. It includes anxiety, depression, ADHD, obsessions, compulsions, addictions, and phobias. Examples include: *“Use of venlafaxine or any other antidepressants, also including St. John's wort within the previous year*” (protocol 24); “*Claustrophobia*” (protocols 5, 10, and 41); “*active suicidal ideation or behavior”* (protocol 43).
4. *Under Guardianship or Legal Order* (22,8%): This category includes individuals under guardianship or those with legal constraints, such as imprisonment. Examples include: *“Loss of ability to freely provide consent through imprisonment or involuntarily incarceration for psychiatric treatment”* (protocol 22); *“Patient under guardianship or deprived of her liberty by a judicial or administrative decision*” (protocol 25).
5. *Not Specified Condition* (5.3%): This cluster captures cases where exclusion criteria were not explicitly defined but were implicitly linked to psychiatric or neurological conditions. Statements such as “*Individuals not able to understand the treatment protocol or sign informed consent*” (protocol 12) or “*lack mental capacity*” (protocol 41) may serve as indirect exclusions for individuals with cognitive or mental impairments.

### 3.3 Analysis of Justifications for Exclusion

Among protocols with exclusion criteria, 70.9% provided justifications, while 29.1% did not. Overall, most exclusions were justified, which is crucial for maintaining transparency and credibility within the process. Justifications for exclusions were categorized into 5 main clusters of concerns regarding exclusion criteria.

1. *Compliance Concern* (39.1%): The most prevalent reason for exclusion, indicating issues related to participants' ability to adhere to study requirements. Examples include: “*Any disorder, which in the Investigator's opinion might jeopardize the participant's safety or compliance with the protocol*” (protocols 8, 12) or “*Patients unwilling to or unable to comply with the protocol*” (protocols 11, 25, 27, 28, 46).
2. *Informed Consent or Inability to Understand* (32,6%): This cluster highlighted worries about participants' capacity to fully comprehend and provide informed consent. Examples include “*Inability to give informed consent*” (protocol 29) and “*Subjects without legal capacity who are unable to understand the nature, scope, significance, and consequences of this clinical trial and to consent*” (protocol 19).
3. *Safety Risks* (13%): Safety risks pertain to prior or concomitant treatments that may compromise participant safety. An example includes “*Any prior or concomitant treatment(s) that might jeopardize the participant's safety*” (protocols 8, 12).
4. *Drug/Product Interference* (10,9%): This concern addresses potential adverse effects on participants and interactions with the study drug. Examples are “*Any medication or condition considered as a contraindication* (…)” (protocol 6) and “*Medication interference*” (protocol 7).
5. *Not in the Best Interest* (4.3%): This cluster reflects considerations regarding participant welfare that extend beyond safety concerns. For example, one protocol stated, “[…] *or is not in the best interest of the participant to participate, in the opinion of the treating investigator*” (protocol 19).

## 4 Discussion

Several analyzed protocols employed exclusion criteria targeting vulnerable populations, often justified by concerns regarding compliance, safety, and the ability to provide informed consent. We propose that excluding these populations from cancer clinical trials may adversely affect the inclusivity and generalizability of clinical research, posing ethical and practical challenges for their representation in cancer studies and broader patient care implications [5]. The following sections will discuss these ethical and practical challenges and their potential effects on clinical trial design and patient outcomes. Additionally, we will examine the influence of heuristic bias on exclusion criteria and outline suggestions for enhancing research inclusivity.

### 4.1 Justifications for Exclusion

The main reported justifications for exclusion in cancer clinical trials include compliance concerns, challenges in obtaining informed consent, drug interference, and safety interests. While often grounded in ethical and legal concerns, such justification for exclusion has significant practical implications that extend beyond individual participant protection [20]. For instance, compliance concerns, frequently mentioned as barriers to inclusion, reflect researchers' apprehensions about maintaining the integrity and validity of trial results, which can be compromised if participants struggle to adhere to study protocols [21, 22]. This concern is particularly emphasized in the case of individuals with mental or neurological conditions, where cognitive impairments might affect understanding or consistent participation [21]. Yet, while compliance concerns are legitimate, they also highlight a critical tension between safeguarding participant welfare and ensuring inclusive and representative research [20, 21].

Challenges in obtaining informed consent represent another justification for exclusion. Individuals with cognitive impairments may face difficulties understanding study requirements, risks, and benefits, which raises ethical complexities in obtaining valid informed consent [10, 21]. Nevertheless, it is important to recognize that excluding individuals ‘under guardianship or judicial order’ may not always be justified. In many cases, legal guardians are authorized to provide informed consent on behalf of these individuals, ensuring that they can still participate in clinical trials. Therefore, automatic exclusion based on guardianship status overlooks the possibility of including these participants with appropriate legal consent, depriving them of access to potentially beneficial research and undermining the inclusivity of the trial.

Different considerations seem to apply instead, in the context of justifications based on safety risks and drug interactions. In these cases, the exclusion highlights the delicate balance between protecting participants and maintaining trial integrity. However, not all protocols provide a clear justification for excluding psychiatric or neurological patients based on the medications they are taking. The lack of further explanation for excluding these patients creates uncertainty about whether the decisions are based on safety concerns, drug interactions, or other reasons, and highlights a potential gap in exploring more nuanced approaches that could balance their inclusion with the need to address associated risks [11, 12, 23].

Furthermore, the results indicate that several protocols do not justify any exclusion, raising concerns about the validity and fairness of the decision‐making process [10]. This absence of explanation can reduce transparency and lead to the exclusion of patients without adequate justification, further exacerbating existing inequities in clinical research. Finally, it is noteworthy to report that, perhaps, the most important document in research ethics, The Helsinki Declaration, states that ‘*The protocol should contain a statement of the ethical considerations involved and should indicate how the principles in this Declaration have been addressed*’, among which inclusion/exclusion criteria and treatment of vulnerable populations are also included ([24], art. 22). Accordingly, we propose that the absence of a detailed rationale for exclusions undermines ethical obligations and may indicate systemic biases against vulnerable populations, emphasizing the need for more inclusive and equitable research practices. Indeed, unsubstantiated exclusions may arise from bias, convenience, or perceived challenges rather than being based on solid evidence of risk or non‐compliance.

### 4.2 Bias and Heuristics in Clinical Trials

Bias can affect the formulation of inclusion and exclusion criteria in clinical trials, resulting in the underrepresentation of specific patient populations [25, 26]. Such bias may arise from assumptions regarding the capacity of individuals with specific conditions to adhere to trial protocols or from overly cautious strategies aimed at managing perceived risks. Consequently, these criteria may inadvertently favor convenience or perceived uniformity over diversity and inclusivity. Bias can manifest in various forms, impacting both the design of the trials and the interpretation of their results. In these cases, the key bias type is the heuristic bias, a mental shortcut that simplifies reasoning, potentially leading to systematic errors [27]. Heuristics are especially employed to make judgments or decisions quickly under conditions of uncertainty. The implications are significant, given that bias can lead to the systemic exclusion of vulnerable populations, thereby limiting the generalizability of research findings and perpetuating disparities in access to potentially beneficial treatments [26]. Yet, these observations highlight the need for more research to confirm the findings and enhance our understanding of their implications.

In clinical research, heuristics might manifest as ‘availability bias’ [28]. This may occur when researchers make decisions based on readily available information or recent experiences rather than a comprehensive assessment. For example, suppose a researcher has recently encountered challenges with participants having psychiatric conditions in a trial. In that case, they might generalize this experience to all participants, leading to broader exclusions than might be warranted. This heuristic can result in over‐cautiousness and potentially unjustified exclusion of individuals who might have been safely included with appropriate safeguards. Another relevant heuristic is the ‘representativeness heuristic’, where researchers might exclude participants based on stereotypes or assumptions about certain conditions [29]. For instance, the assumption that all individuals with psychiatric or neurological conditions will struggle with compliance or understanding the study protocol can lead to blanket exclusions. This heuristic may overlook the variability within these populations and the potential for adaptive strategies that could facilitate their participation. Finally, ‘status quo bias’ may also impact exclusion practices [30]. This type of bias favors the preservation of existing conditions or methods over embracing new approaches or changes. In clinical trials, such bias can lead researchers to adhere to traditional exclusion criteria and practices, or even to replicate outdated protocols, despite the availability of alternative approaches that could effectively address safety concerns and promote inclusivity.

To mitigate heuristic biases, researchers might implement structured decision‐making processes and incorporate diverse perspectives into trial design. Engaging with experts and individuals with lived experience can help counteract biases and ensure that exclusion criteria are informed by a comprehensive, evidence‐based assessment. Furthermore, adopting flexible trial designs, such as adaptive trials, and improving informed consent procedures can better accommodate diverse participant needs, enhancing the inclusivity and relevance of clinical research. The following section will explore further strategies to address these and additional challenges.

### 4.3 Clinical Implications

As stated, the reference policy document in research ethics is represented by the Helsinki Declaration [24]. Despite being originally devised only as a soft law, the Helsinki Declaration is the only document mentioned in the Clinical Trial Regulation and its dictates have thus become binding for clinical experimenters and investigators. The Helsinki Declaration has devoted two articles to the treatment of vulnerable populations and groups. The Declaration states that the inclusion of vulnerable populations in research should be subjected to the fulfillment of a threefold requirement: “*(i) if it is responsive to their health needs and priorities; […] (ii) the individual, group, or community stands to benefit from the resulting knowledge, practices, or interventions; […] (iii) when the research cannot be carried out in a less vulnerable group or community, or when excluding them would perpetuate or exacerbate their disparities*” ([24], art. 20). Overall, these criteria indicate that while the inclusion of vulnerable populations must be approached with care, one of the key reasons for their inclusion is to prevent the continuation of disparities affecting these groups. In other words, “*the harms of exclusion must be considered and weighed against the harms of inclusion*” ([23], art. 19). Therefore, the approach detailed in the recent reformulation of the Declaration [24] appears to strike a balance between its traditional protective stance and the growing emphasis on fairness and inclusion. This aligns with trends in academic bioethics, which advocate for the careful inclusion of vulnerable populations (e.g., [8, 14, 20, 31]).

However, it is crucial to recognize that cancer individuals, who are undergoing significant mental and emotional distress as a direct result of their diagnosis and treatment, are often ordinary people struggling with the side effects of their illness. Psychiatric diagnoses among cancer patients are common and include trauma or stress‐related conditions, anxiety, sleep disturbances, depression, and other mood disorders [32, 33]. Neurocognitive issues, adjustment disorders, and somatic symptoms are also prevalent, as well as psychosis, which can be linked to paraneoplastic syndromes or neuroplastic lesions [34, 35]. Excluding individuals from potentially life‐saving clinical trials based on their condition, could deny them access to crucial research opportunities and effective interventions. Such exclusions can not only restrict access to crucial treatments but also worsen disparities in research opportunities [10, 11, 12, 13, 36]. Bridging this gap needs a thoughtful approach that acknowledges the specific needs of these populations while maintaining strong ethical standards [8, 14, 31, 37]. Creating clear, evidence‐based guidelines for including individuals with these conditions can help balance ethical concerns with the aim of inclusiveness in research. Although ethical guidelines provide criteria for excluding vulnerable populations when needed, they may result in blanket exclusions and missing chances for adaptive trial designs that could safely include these participants with proper safeguards.

To address disparities in clinical trials, we recommend adopting inclusive recruitment strategies and flexible trial designs. Adaptive trials, which modify protocols based on real‐time data, provide a viable method for balancing participant safety with inclusivity [38, 39]. Enhancing informed consent procedures with simplified language, decision aids, and supportive frameworks can help individuals with cognitive impairments, as many can still communicate their willingness to participate [40, 41]. To further ensure ethical inclusion, implementing periodic capacity reassessments and utilizing tailored consent processes (e.g., staged consent or supported decision‐making) can help accommodate fluctuating cognitive abilities. Additionally, engaging patient advocacy groups and including representatives of vulnerable populations in trial design may ensure that exclusion criteria are carefully considered. Researchers also may hesitate to include vulnerable populations due to uncertainty about the definition and scope of vulnerability, highlighting the need for standardized guidance and targeted training. Providing concrete tools and educational initiatives could help researchers navigate ethical and methodological challenges, promoting more inclusive research practices. Suggesting strategies such as stratified study designs, enhanced monitoring, and individualized dosages can create alternative pathways for participation as well [41]. Furthermore, incorporating ethics consultation services within trial oversight committees may assist researchers in making case‐by‐case decisions about inclusion, together with obtaining consent through legal guardians or using simplified processes to facilitate the involvement of those typically excluded.

Finally, as a broader step forward, we suggest a tiered accountability approach that combines mandatory justification for exclusion criteria—particularly when involving historically underrepresented populations—with inclusion planning during protocol development. In addition, implementing monitoring mechanisms to benchmark inclusion across trials could encourage more equitable practices without imposing rigid quotas, fostering a culture of reflective and inclusive research design. To conclude, adaptive approaches would broaden access to clinical trials and generate more representative data, ultimately leading to more inclusive and applicable evidence for all cancer patients.

### 4.4 Limitations

This study presents some limitations. Its focus on specific cancer types within the EU Clinical Trials Register may restrict the generalizability of the findings to other cancers or regions. Moreover, the study does not compare data from the EU Clinical Trials Register with other global trial databases, such as ClinicalTrials.gov, which could have provided additional insights and a broader context. Finally, it relies on protocol documentation, which may not fully capture real‐world exclusion decisions or adjustments made during the trials. Despite these constraints, the study offers critical insights into exclusion practices in cancer trials.

## 5 Conclusions

The current study reveals significant gaps in the inclusion of vulnerable populations in cancer clinical trials, primarily due to exclusion criteria related to compliance, safety, and informed consent. While these exclusions are often justified, they highlight the need for a more inclusive trial design that balances participant protection with the ethical imperative to enhance research participation. Ethical guidelines, such as those in the Helsinki Declaration, aim to protect vulnerable individuals but can inadvertently perpetuate healthcare disparities by denying these groups access to potentially beneficial trials. Such exclusions narrow the evidence based on the efficacy and safety of treatments for these populations and hinder the development of tailored therapeutic approaches that meet their specific healthcare needs. To foster more equitable representation in cancer trials, the scientific community should establish clear guidelines, employ adaptive trial designs, and enhance informed consent processes. This will strengthen the evidence base for treating cancer across diverse populations, ultimately leading to more personalized and effective care.

## Author Contributions

**Margherita Dahò:** conceptualization, data curation, formal analysis, investigation, methodology, validation, visualization, writing – original draft, writing – review and editing. **Veronica Coppini:** conceptualization, data curation, investigation, methodology, writing – original draft, writing – review and editing. **Maria Vittoria Ferrari:** conceptualization, data curation, investigation, methodology, writing – original draft, writing – review and editing. **Giulia Ferraris:** conceptualization, data curation, investigation, methodology, writing – review and editing. **Virginia Sanchini:** conceptualization, methodology, supervision, validation, writing – original draft, writing – review and editing. **Dario Monzani:** conceptualization, data curation, methodology, validation, supervision, writing – review and editing. **Roberto Grasso:** conceptualization, methodology, supervision, writing – review and editing. **Chiara Agnello:** supervision, writing – review and editing. **Giuseppe Badalamenti:** supervision, writing – review and editing. **Laura Algeri:** supervision, writing – review and editing. **Gabriella Pravettoni:** conceptualization, methodology, funding acquisition, project administration, resources, supervision, validation, visualization, writing – original draft, writing – review and editing.

## Ethics Statement

This study did not involve human participants; therefore, no ethical approval was required.

## Conflicts of Interest

The authors declare that the research was conducted in the absence of any commercial or financial relationships that could be construed as a potential conflict of interest.

## Supporting information

Table S1

## Data Availability

The data supporting the findings of this study are available in the supplementary material provided with this manuscript.
