# Supplementary material for: Exploring Barriers to Inclusivity: Systematic Analysis of Exclusion Criteria and Potential Bias in Clinical Cancer Trials for Psychiatric and Neurological Conditions in European Protocols
Source: Psychooncology. 2025 May 12;34(5):e70182. doi: 10.1002/pon.70182 (PMC12068421; doi:10.1002/pon.70182)
Supplement: Supplementary file 1 — Table S1 [file PON-34-e70182-s001.docx]

**Supplementary File

Table A. Overview of Exclusion Criteria Related to Psychiatric and Neurological Conditions in Cancer Clinical Trials**

This table provides a summary of exclusion criteria applied to clinical cancer trials that specifically relate to psychiatric and neurological conditions. It includes the code number we gave to each protocol, the type of cancer under investigation, the exclusion criteria involving psychiatric or neurological conditions, detailed descriptions of these exclusions, and the justifications provided for their implementation.

| **Protocol number** | **Type of Cancer** | **Exclusion criteria with Psychiatric/ neurological condition** | **Detail of exclusion** | **Justification** | **Justification of exclusion** |
| --- | --- | --- | --- | --- | --- |
|  | Prostate | Yes | Patients with neurological diseases; such as CVA, TIA, Parkinson, and polyneuropathy. | No | No |
|  | Prostate | No | N/A | N/A | N/A |
|  | Prostate | Yes | Patients with neurological disease, psychiatric disease, drug or alcohol abuse. | Yes | These diseases could interfere with the participant’s proper compliance. |
|  | Prostate | Yes | Patients with history of relevant CNS pathology or current relevant CNS pathology (e.g. seizure, paresis, aphasia, cerebrovascular ischemia/hemorrhage, severe brain injuries, dementia, Parkinson’s disease, cerebellar disease, organic brain syndrome, psychosis, coordination or movement disorder) | No | No |
|  | Prostate | Yes | Claustrophobia | No | No  (probabilmente però per paura della MRI) |
|  | Prostate | No | Any medication or condition considered as a contraindication to estradiol (allergy to adjuvant compounds (carbomer, trolamine), history with thromboembolic disorders (protein C, protein S, or antithrombin deficiency), porphyria, acute or previous liver disease, drugs with cytochrome P450 enzyme metabolism (anticonvulsants: phenobarbital, phenytoin, carbamazepine; anti-infectives: rifampicin, rifapentine, nevirapine, efavirenz; and St. John's wort)) or leuprorelin (allergy to adjuvant compounds (polylactic acid), Qt-time prolonging drugs (quinidine, disopyramide, amiodarone, sotalol, dofetilide, ibutilide, methadone, moxifloxacin, antipsychotics) | No | No |
|  | Prostate | Yes | Patients receiving opioid substitution therapy for opioid addiction (e.g. methadone or buprenorphine) and/or suffering from opioid withdrawal. | Yes | Medication interference |
|  | Prostate | Yes | Not Specified | Yes | Any prior or concomitant treatment(s) that might jeopardise the participant’s safety or that would compromise the integrity of the Trial.  Any disorder, which in the Investigator’s opinion might jeopardise the participant’s safety or compliance with the protocol; not able to understand the treatment protocol or sign informed consent. |
|  | Prostate | No | N/A | N/A | N/A |
|  | Prostate | Yes | Claustrophobia | Yes | Inability to undergo MRI |
|  | Prostate | Yes | Not specified | Yes | Any condition that makes the patient unable to comply with study procedures or unwillingness to participate in the study. |
|  | Prostate | Yes | Not specified | Yes | Any prior or concomitant treatment(s) that might jeopardise the participant’s safety or that would compromise the integrity of the Trial  Any disorder, which in the Investigator’s opinion might jeopardise the participant’s safety or compliance with the protocol.  Individuals not able to understand the treatment protocol or sign informed consent. |
|  | Prostate | Yes | Current relevant CNS pathology (e.g. seizure, paresis, aphasia, cerebrovascular ischemia/ hemorrhage, severe brain injuries, dementia, Parkinson’s disease, cerebellar disease, organic brain syndrome, psychosis, coordination or movement disorder) | No | No |
|  | Breast | Yes | Patients with a history of uncontrolled seizures, central nervous system disorders or psychiatric disability judged by the investigator to be clinically significant precluding study participation. | Yes | [...] disability judged by the investigator to be clinically significant precluding study participation. |
|  | Breast | Yes | Cognitive function impairment | Yes | Subjects that for some reason are unable to exercise their rights, such as cognitive function impairment. |
|  | Breast | No | N/A | N/A | N/A |
|  | Breast | No | N/A | N/A | N/A |
|  | Breast | No | N/A | N/A | N/A |
|  | Breast | Yes | 16.  Has known psychiatric or substance abuse disorders that would interfere with cooperation with the requirements of the trial. | Yes | [...] Any condition, therapy, or laboratory abnormality that might confound the results of the study, interfere with the participant’s participation for the full duration of the study, or is not in the best interest of the participant to participate, in the opinion of the treating investigator. […]  Disorders that would interfere with cooperation with the requirements of the trial.  Subjects without legal capacity who are unable to understand the nature, scope, significance and consequences of this clinical trial and to consent. |
|  | Breast | Yes | Not specified | Yes | Inability to understand the nature, risks, and benefits of the study |
|  | Breast | No | N/A | N/A | N/A |
|  | Breast | Yes | treatment of either a psychiatric or physical illness  Patients may be discontinued from the study therapy in the following circumstances: Loss of ability to freely provide consent through imprisonment or involuntarily incarceration for treatment of either a psychiatric or physical (e.g., infectious disease) illness. | Yes | Patients will be considered to have not completed the study if they withdraw from the study for any of the following reasons: - Other (protocol deviation, technical problems, loss of ability to freely provide consent through imprisonment or involuntarily incarceration for treatment of either a psychiatric or physical illness). |
|  | Breast | Yes | 8. Other severe acute and/ or chronic medical and/ or psychiatric condition and/ or laboratory abnormality that would impart, in the judgment of the Investigator, risk associated with study participation or (N)IMP administration, or which, in the judgment of the Investigator, would make the patient inappropriate for participation in this study. 20.  Patients with uncontrolled seizures | Yes | [...] Any abnormality that would impart, in the judgment of the Investigator, risk associated with study participation or (N)IMP administration, or which, in the judgment of the Investigator, would make the patient inappropriate for participation in this study |
|  | Breast | Yes | Use of venlafaxine or any other antidepressants, also including St. John's wort within the previous year;  Use of gabapentin and/or calcium channel antagonists within 2 weeks of study entry; Simultaneous use of Monoamine oxidase inhibitors (MAOIs). | No | No |
|  | Breast | Yes | 16. Psychological, familial, sociological or geographical conditions that do not permit compliance with the study protocol. 20.  Patients unwilling to or unable (as assessed by the investigator) to comply with the protocol. 21.  Patient under guardianship or deprived of her liberty by a judicial or administrative decision or incapable of giving its consent. | Yes | 20.  Patients unwilling to or unable (as assessed by the investigator) to comply with the protocol. 21.  Patient under guardianship or deprived of her liberty by a judicial or administrative decision or incapable of giving its consent  [...] Conditions that do not permit compliance with the study protocol. Patients unwilling to or unable (as assessed by the investigator) to comply with the protocol [...] or incapable of giving its consent. |
|  | Breast | Yes | History of significant neurological or psychiatric disorders including psychotic disorders, dementia or seizures that would prohibit the  understanding and giving of informed consent. | Yes | [conditions] that would prohibit the understanding and giving of informed consent. |
|  | Breast | Yes | Not specified | Yes | Not able to understand and to comply with study instructions and requirements. |
|  | Breast | Yes | Not specified | Yes | 20.  History of non-compliance to medical regimens.  21.  Unwilling or unable to comply with the protocol. |
|  | Breast | Yes | The drugs listed below should be avoided, because some of these drugs could alter the way  in which Indocyanine Green, is absorbed into the body, and could make the diagnosis  inaccurate:  - Anticonvulsants  - Cyclopropane  - Bisulphite compounds (conservative product)  - Haloperidol (antipsicotico)  - Heparine  - Diamorphine  - Pethidine  - Metamizole  - Methadone  - Morphine  - Nitrofurantoin  - Opium alkaloids  - Phenobarbital (usato anche per ansia, insomnia)  - Phenylbutazone  - Probenecid  - Rifamycin  - Sodium iodide i-123 | Yes | 14.  Inability to give informed consent  drugs could alter the way in which Indocyanine Green, is absorbed into the body |
|  | Breast | Yes | 20.  Any severe, acute, uncontrolled, or chronic medical or psychiatric condition or laboratory abnormality that may increase the risk associated with study participation or investigational or non-investigational products administration, or may interfere with the interpretation of study results, and, in the judgment of the investigator, would make the patient inappropriate for entry into this study. Moreover, patients who, by virtue of an order issued by judicial or administrative authorities, are committed to an institution or those who cannot take part in clinical trials are excluded from this study.  21.  History of significant neurological or psychiatric disorders including psychotic disorders, dementia, or seizures that would prohibit the understanding and giving of informed consent. Also, prior history of posterior reversible encephalopathy syndrome (PRES) excludes patients from this study.  22.  Unable or unwilling to avoid medications, supplements (e.g., St. John’s wort), or foods (e.g., grapefruit, pomegranate, pomelos, star fruit, Seville oranges and their juices) that are moderate/strong inhibitors or inducers of CYP3A4 activity. Participation will be allowed if the medication, supplements, or foods are discontinued for at least 14 days prior to study entry and for the duration of the study. | Yes | [Any] abnormality that may increase the risk associated with study participation or investigational or non-investigational products administration, or may interfere with the interpretation of study results, and, in the judgment of the investigator, would make the patient inappropriate for entry into this study  [or that] would prohibit the understanding and giving of informed consent [or that would make the patient] unable or unwilling to avoid medications [...] or foods [...] that are moderate/strong inhibitors or inducers of CYP3A4 activity. |
|  | Breast | Yes | Nervous system disorder | No | No |
|  | Breast | Yes | Exclusion criteria: Uncontrolled hypertension, heart, liver, kidney related or other medical or psychiatric disorders. | No | No |
|  | Breast | Yes | Exclusion criteria: Inability to cooperate.  Alcohol and/or drug overuse (investigators decision). | Yes | Inability to cooperate. |
|  | Breast | Yes | Co-administration of giredestrant with the following concomitant therapies should be avoided:  · Strong CYP3A inhibitors, including, but not limited to, the following: atazanavir, ritonavir,  lopinavir, telaprevir, telithomycin, indinavir, nelfinavir, saquinavir, clarithromycin,  troleandomycin, itraconazole, ketoconazole, voriconazole, posaconazole, nefazodone,  mibefradil  · Strong CYP3A inducers, including, but not limited to, the following: apalutamide rifampin,  carbamazepine, phenytoin, enzalutamide, lumacaftor, and hyperforin (St. John's Wort).  The above lists of CYP3A concomitant medications are not necessarily comprehensive. | Yes | Patient has any other concurrent severe and/or uncontrolled medical condition that would, in the Investigator’ opinion cause unacceptable safety risks, contraindicate patient participation in the clinical trial or compromise compliance with the protocol.  Patient has a history of non-compliance to medical regimen. |
|  | Breast | Yes | 5. History of alcohol abuse. 20.  Medical or psychiatric comorbidities rendering the patient not candidate to the clinical trial, according to the investigator’s judgement. | Yes | [...] comorbidities rendering the patient not candidate to the clinical trial, according to the investigator’s judgement. (è un problema di compliance?) |
|  | Breast | Yes | 2.Contraindications according to SmPC for the CDK 4/6-inhibitors that is  planned to be used. Specifically, any hypersensitivity to the active substance or  to any of the excipients or to peanut, soya (for ribociclib) or use of preparations  containing St. John’s Wort (for palbociclib) are contraindications. | Yes | Unable to give informed consent |
|  | Breast | No | N/A | N/A | N/A |
|  | Lung | No | N/A | N/A | N/A |
|  | Lung | Yes | Not specified | Yes | Subject is not able and willing to sign the Informed Consent Form |
|  | Lung | Yes | Not specified | Yes | Unable to comply with the protocol, including acceptable candidacy for adjuvant chemotherapy according to local institutional standards and likely compliance with follow-up for anticipated length of study (i.e. 5 years from the initiation of enrollment). |
|  | Lung | Yes | 1. (Anticipated) inability to complete the breath sampling procedure (e.g., inability to maintain  adequate ventilation unaided or claustrophobia).  2. Potential subjects if in the opinion of the investigator lack mental capacity | Yes | (Anticipated) inability to complete the breath sampling procedure (e.g., inability to maintain adequate ventilation).  Addition of participants lacking mental capacity to exclusion criteria to bring the protocol in line with the UK protocol. |
|  | Lung | No | N/A | N/A | N/A |
|  | Lung | Yes | 7. Patients with symptomatic or neurologically unstable CNS metastases.  15. Other severe acute or chronic medical or psychiatric conditions, including recent (within the past year) or active suicidal ideation or behavior, or laboratory abnormality that may increase the risk associated with study participation or investigational product administration. or may interfere with the interpretation of study results and, in the judgment of the investigator, would make the patient inappropriate for entry into this study. | Yes | Any abnormality that may increase the risk associated with study participation or investigational product administration or may interfere with the interpretation of study results and, in the judgment of the investigator, would make the patient inappropriate for entry into this study. |
|  | Lung | Yes | Mental or psychological illness that does not allow the patient to give informed consent. | Yes | Inability of the patient to give informed consent. |
|  | Lung | No | It is known there is a potential for palbociclib to increase the exposure of axitinib through a time-dependent CYP3A4 mediated drug-drug interaction. As a weak time-dependent CYP3A4 inhibitor, palbociclib may reduce the clearance of axitinib, which may increase its exposure-related toxicities. Therefore, it is advised to: Exclude patients currently taking strong CYP3A4 inducers and inhibitors if an alternative concomitant medication cannot be.  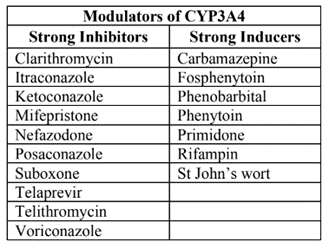 | N/A | As a weak time-dependent CYP3A4 inhibitor, palbociclib may reduce the clearance of axitinib, which may increase its exposure-related toxicities. Therefore, it is advised to: Exclude patients currently taking strong CYP3A4 inducers and inhibitors if an alternative concomitant medication cannot be. |
|  | Lung | Yes | 2. Subjects with symptomatic CNS metastases or leptomeningeal disease who are neurologically unstable or have required increasing doses of steroids to manage CNS symptoms within the 2 weeks prior to study day  15. Judgment by the investigator that the subject should not participate in the study if the subject is unlikely to comply with study procedures, restrictions and requirements.  9. Use of known cytochrome P450 (CYP) 3A4 sensitive substrates (with a narrow therapeutic  window), within 14 days or 5 half-lives of the drug or its major active metabolite, whichever is  longer, prior to study day 1 that was not reviewed and approved by the principal investigator.  Use of strong inducers of CYP3A4 (including herbal supplements such as St. John’s wort) within 14 days or 5 half-lives (whichever is longer) prior to study day 1 that was not reviewed and approved by the principal investigator. | Yes | The subject is unlikely to comply with study procedures, restrictions and requirements.  Neurologically unstable brain metastases. |
|  | Breast | Not English written |  |  |  |
|  | Breast | Impossible download |  |  |  |
|  | Breast | Impossible download |  |  |  |
|  | Lung | Impossible download |  |  |  |
|  | Prostate | Not English written |  |  |  |
